# Seeking motivation and reward: Roles of dopamine, hippocampus, and supramammillo-septal pathway

**DOI:** 10.1016/j.pneurobio.2022.102252

**Published:** 2022-02-25

**Authors:** Andrew J. Kesner, Coleman B. Calva, Satoshi Ikemoto

**Affiliations:** a Laboratory for Integrative Neuroscience, Division of Intramural Clinical and Biological Research, National Institute on Alcohol Abuse and Alcoholism, NIH, Bethesda, MD, USA; b Center on Compulsive Behaviors, Intramural Research Program, NIH, Bethesda, MD, USA; c Behavioral Neuroscience Research Branch, Intramural Research Program, National Institute on Drug Abuse, NIH, Baltimore, MD, USA

**Keywords:** Music perception, Risk taking, Reward prediction error, Expectancy, Latent learning, Stimulus-stimulus learning

## Abstract

Reinforcement learning and goal-seeking behavior are thought to be mediated by midbrain dopamine neurons. However, little is known about neural substrates of curiosity and exploratory behavior, which occur in the absence of clear goal or reward. This is despite behavioral scientists having long suggested that curiosity and exploratory behaviors are regulated by an innate drive. We refer to such behavior as information-seeking behavior and propose 1) key neural substrates and 2) the concept of environment prediction error as a framework to understand information-seeking processes. The cognitive aspect of information-seeking behavior, including the perception of salience and uncertainty, involves, in part, the pathways from the posterior hypothalamic supramammillary region to the hippocampal formation. The vigor of such behavior is modulated by the following: supramammillary glutamatergic neurons; their projections to medial septal glutamatergic neurons; and the projections of medial septal glutamatergic neurons to ventral tegmental dopaminergic neurons. Phasic responses of dopaminergic neurons are characterized as signaling potentially important stimuli rather than rewards. This paper describes how novel stimuli and uncertainty trigger seeking motivation and how these neural substrates modulate information-seeking behavior.

*Anticipation of pleasure is, in itself, a very considerable pleasure.* DAVID HUME, *A Treatise of Human Nature*.

A mind is fundamentally an anticipator, an expectation-generator. It mines the present for clues, which it refines with the help of the materials it has saved from the past, turning them into anticipations of the future.

DANIEL DENNETT, *Kinds of Minds*.

## 1. Introduction

**Fig. 1.**
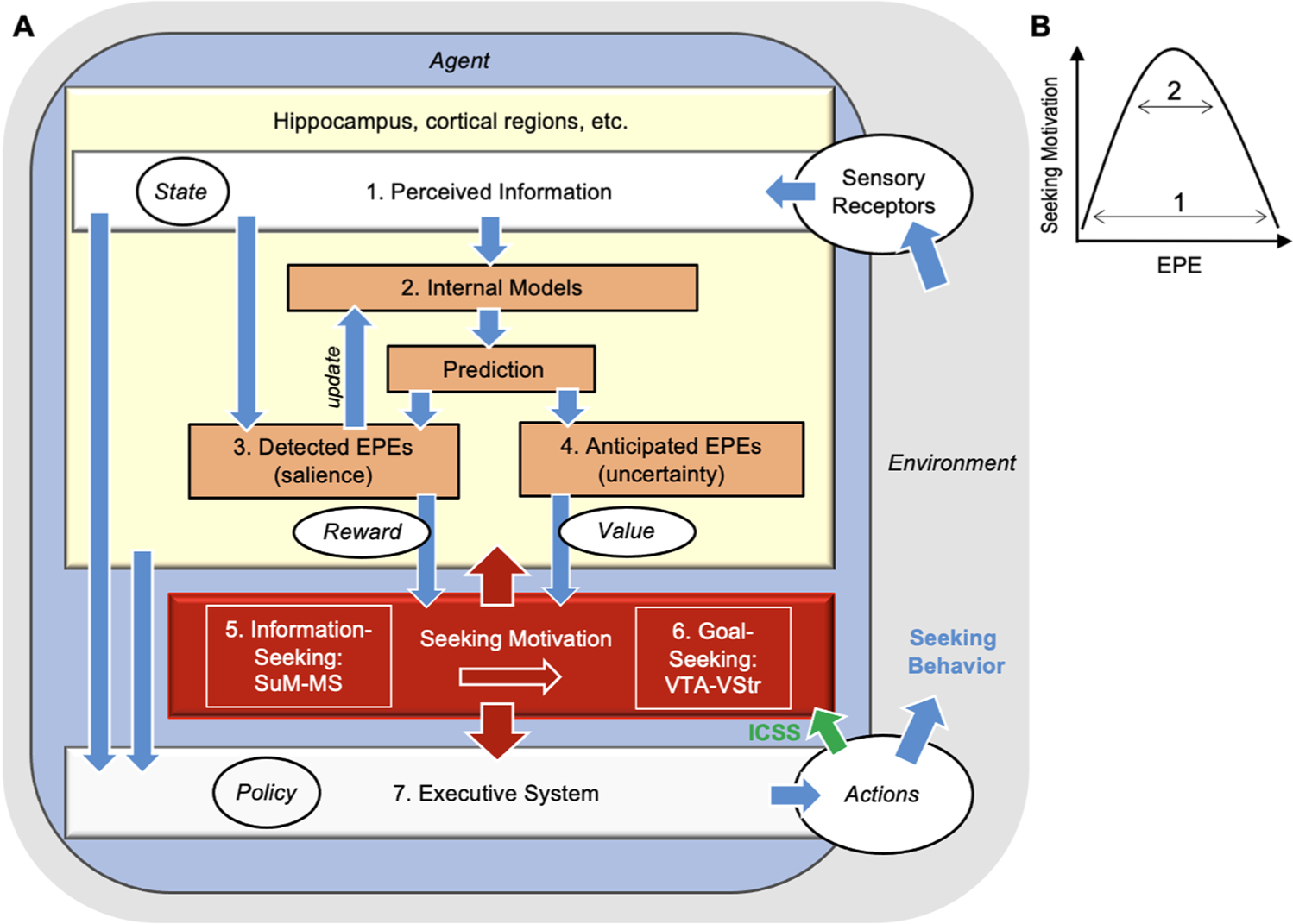
A conceptual model for seeking behavior process. A. The diagram shows a highly simplified model describing relationships among key concepts related to seeking motivation (see Sections 1 and 2). Although it is not straightforward to characterize a neurobiology-based model in terms of the concepts used in machine or reinforcement learning, reinforcement learning concepts (Sutton and Barto, 1998) are shown in italic in the proposed model, to clarify the relationships among key concepts: *agent*, the learner and decision maker; *environment*, things that interact with the agent; *state*, anything that the agent have observed (could include the Internal Models); *policy*, the rule that the agent employ selective action options; *reward*, experienced goal (i.e., optimal level of detected EPE); *value*, desired goal (i.e., optimal level of anticipated EPE); *action*, selected behavioral response. The goal of the model is to maximize knowledge about the environment. The process executes as follows: (1) The agent or animal perceives the environment (*state*). (2) The perceived information is compared against the prediction generated by internal models. (3) The comparison results in the detection of environment prediction errors (EPEs). Detected EPEs are integrated into the models, to update knowledge, which generate new predictions. (4) New predictions are subjectively assessed for their likelihood, generating future, anticipated EPEs (i.e., uncertainty). (5) Detected and anticipated EPEs are used to produce reward and value, respectively, as shown in (B; Although reward and value are distinguished in the model due to being differentially derived, both are motivators. See subsection 1.1). Reward/value activates the information-seeking process, depending on the supramammillo-septal pathway (SuM-MS). (6) The information-seeking process modulates the activity of the goal-seeking process, depending on the meso-limbic pathway (VTA-VStr). (7) The executive system produces *actions* based on perceptual and cognitive inputs, motivation inputs, and *policy*, which partly comes from the internal models. This process is repeated until the animal gets “bored” caused by low or high EPEs (B) or until seeking behavior is interrupted by other needs, including rewards^classic^, threats, fatigue, etc. In addition, the diagram explains how intracranial self-stimulation (ICSS) is produced. Artificial activation of certain neural elements of seeking motivation (green arrows) activates the executive system, which instigates seeking response, and if the seeking response is contingently paired with such activation, animals display ICSS. B. EPE level has an inverted U-curve relationship with seeking motivation level. The information-seeking process may be affected by broad range of EPEs (1), while the goal-seeking process may be responsive to a narrow EPE range (2).

The ability to anticipate future events provides survival advantage. Human and non-human animals who can anticipate the future can prepare themselves for opportunities and dangers. The anticipation of future events depends on remembering the past and perceiving the present. Thus, animals need to acquire environmental information and store it for future use. To this end, an animal’s active interaction with its environment that leads to information acquisition would help the animal generate the best possible prediction of future events. We discuss the existence of a motivation system that coordinates cognition and behavior to acquire information and suggest neural substrates of these processes. In addition, we discuss how midbrain dopamine neurons are involved in acquiring environmental information. Our ideas are summarized in Fig. 1, which is explained over multiple, upcoming sections.

### 1.1. Reward and behavior

Behavior is motivated by reward. This term, reward, is defined as having behavioral effect without referring to any conscious experience as follows: A reward is a thing, experience, or brain manipulation that instigates and reinforces approach behavior. Therefore, *if a stimulus or event instigates and reinforces approach behavior, then it is a reward*.

Accordingly, rewards can be any of a variety of things, including food, water, novel stimuli, abused substances, and intracranial manipulations. We distinguish classic rewards (rewards^classic^) such as food and water from non-conventional rewards which are discussed below. Rewards^classic^ such as food and water are regulated homeostatically. Homeostatic needs instigate animals to seek rewards^classic^ or conditioned stimuli that have been associated with rewards^classic^ (CS^reward^). This results in CS^reward^ acquiring motivational properties of rewards^classic^ and thereby becoming important guides for seeking behaviors. Such behavior persists until homeostatic needs are met, and therefore, are typically labeled as goal-directed behavior or goal-seeking behavior. In addition, certain species-specific stimuli, including reproduction-related stimuli and other social stimuli, are homeostatically regulated (Matthews and Tye, 2019). For example, social play behavior in juvenile rats serves as a reward and is regulated by age and exposure amount (Ikemoto and Panksepp, 1992; Panksepp, 1981b; Panksepp and Beatty, 1980; Trezza et al., 2011). Therefore, they can instigate goal-seeking behavior.

The other class of rewards is environmental stimuli that do not have a direct link with biological needs but can be characterized as salient or novel stimuli. Although many such stimuli can instigate and reinforce approach behavior, their effects as such are transient and not as persistent as those of rewards^classic^ and CS^reward^. In other words, they are not considered to be behavioral goals, but instigators of curiosity and exploratory behavior. Therefore, we refer to the behavioral interaction with such stimuli as *information-seeking behavior*.

Note that artificial rewards such as abused substances (e.g., cocaine and heroin) and brain stimulation rewards (e.g., intracranial self-stimulation, see subsection 2.7) are not classified as rewards^classic^ because no evidence indicates that they are regulated by homeostatic or specialized, evolutionarily selected mechanisms. However, they can elicit powerful reinforcing effects such that animals and humans seek them despite negative consequences (Koob and Volkow, 2010; Olds, 1958; Routtenberg and Lindy, 1965). The reason for this, at least in part, is that they can activate midbrain dopamine neurons (Koob and Volkow, 2010; Wise and Bozarth, 1987), which play a critical role in goal-seeking behavior (Fig. 1A; see Section 7).

In sum, sensory stimuli associated with rewards^classic^ can act as persistent goals and support goal-seeking behavior. Alternately, other environmental stimuli can serve as rewards whose effects are transient and inconsistent, and support information-seeking behavior.

### 1.2. Information-seeking behavior

Curiosity and exploratory behavior are suggested to be regulated by a *drive* or motivation system (Dashiell, 1925; Nissen, 1930), and it is reinforcing to engage in the action to satisfy or activate that motivation (Berlyne, 1950; Montgomery, 1954; Woodworth, 1958). For example, rodents learn operant responding for the opportunity to explore an environment that contains no rewards^classic^ (Montgomery, 1954; Myers and Miller, 1954). Similarly, monkeys learn operant responding for the opportunity to view their surroundings from an enclosed chamber (Butler, 1953; Butler and Harlow, 1954). The existence of such motivation is further supported by the phenomenon called latent learning (Blodgett, 1929; Tolman, 1948). For example, Tolman and his colleagues famously demonstrated that rats left in a maze with multiple paths and corners in the absence of any reward^classic^ still learn about maze environments. This knowledge is demonstrated by efficient seeking behavior when rewards^classic^ are later introduced in the maze (Tolman, 1948). These behavioral observations support motivational processes that instigate and reinforce information-seeking behavior in the absence of rewards^classic^. We now refer to the processes as *seeking motivation*, and we suggest that this motivation also plays an important role in the seeking for rewards^classic^. Note that the term motivation is used here in two ways: (1) a coordinator of multiple structural and sub-functional activities for specified function and (2) an invigorator that increases occurrences or effort for specified function.

In the ensuing sections, we will provide further evidence and elaborate on how this motivation system coordinates neural substrates to regulate seeking behavior for environmental information (i.e., information-seeking behavior) and rewards^classic^ (i.e., goal-seeking behavior). Other groups have also begun investigating how the brain regulates seeking behavior that is not necessarily linked directly with rewards^classic^ (Ahmadlou et al., 2021; Bromberg-Martin and Hikosaka, 2009; Daddaoua et al., 2016; Gottlieb et al., 2014; Gruber and Ranganath, 2019; Marvin et al., 2020; Monosov, 2020). This review also discusses how the proposed mechanisms of seeking motivation relate to the midbrain dopamine (DA) system. DA neurons are known to display phasic activities that are characterized as reward prediction errors (RPEs), the difference between actual reward^classic^ and predictions of the *time* and *magnitude* of reward^classic^, to teach the animal relationships between the environment, behavior, reward^classic^, and punishment and to shape goal-seeking behavior (Schultz et al., 1997). Note that in addition to reinforcement learning, phasic signals can produce motivation. Phasic DA activity is correlated with reward-seeking responses (Phillips et al., 2003; Roitman et al., 2004; Satoh et al., 2003), and phasic stimulation of DA neurons or medial prefrontal cortex neurons (which in turn activate midbrain DA neurons (Beier et al., 2015)) is found to produce invigorating effects on reward-seeking responses (Hamid et al., 2016; Fig. 6 of Ilango et al., 2014a; Fig. 2f of Yang et al., 2022).

## 2. Environment and seeking motivation

### 2.1. Environment prediction error

To facilitate mechanistic discussions of information-seeking behavior, we propose the term *environment prediction errors* (EPEs). EPEs allow the animal to learn about the environment and motivate the animal to seek environmental information (Fig. 1). First, EPEs are used to update internal models of the environment. Internal models of the environment are developed over time by acquiring previously perceived information and, in turn, generate predictions about the environment. The prediction is compared against perceived environment, and the discrepancy between the prediction and the perception concerning the environment is an EPE. Second, although it may not be intuitive, EPEs will invigorate the animal to seek more information (see subsection 2.3 for evidence). Detected EPEs are associated with such perceptions as *novelty* and *salience*. Novel and salient stimuli are worthy of attention and investigation because such perceptions indicate that their features are not clearly represented in the internal models.

An important, related term is *uncertainty*. In terms of EPE, uncertainty is a subjective confidence level about the prediction that the internal model has generated and not yet been evaluated, thereby the anticipation of EPEs. Like detected EPEs (i.e., novelty and salience), anticipated EPEs (i.e., uncertainty) instigate attention and investigation to learn more about the environment.

In summary, both detection and anticipation of EPEs depend on internal models, which have been developed through prior environmental interactions with sensation and perception, learning and memory, and integration of such processes. Because acquired information should make it more effective in predicting the future for obtaining rewards^classic^ and avoiding dangers, the brain must be organized in such a way to detect and anticipate EPEs and then to instigate information-seeking behavior to update internal models.

### 2.2. Seeking motivation

Seeking motivation coordinates cognitive and behavioral processes to produce adaptive seeking behavior. For one, it increases the cognitive capacity for attending and perceiving environmental information, storing it for future use, and recalling it. Thus, seeking motivation has a positive feedback relation with cognition (Fig. 1A). In addition, seeking motivation invigorates approach behavioral responses toward the environment. Note that approach behavior includes active avoidance behavior, which is a type of approach behavior - approach toward safety (see subsection 7.2). For example, hearing fire alarm in a high-rise building instigates seeking behavior for a staircase. This seeking behavior is active in the same way that, for example, the sight of a novel object in a familiar environment instigates seeking behavior toward the object. Such actions are associated with such feelings as hope, desire, and energy. Thus, active avoidance behavior should be distinguished from passive avoidance (i.e., freezing) behavior. The detection of a predator in proximity will elicit passive avoidance behavior, which is associated with fear, anxiety, and aversion. Negative affective states at extreme levels elicit escape behavior, which occurs in the absence of seeking motivation. Thus, EPEs can be positive and instigate active seeking behavior, or negative and elicit passive avoidance or escape behaviors. The present paper focuses on seeking motivation that instigates approach, seeking behavior

### 2.3. Relationship between seeking motivation and learning

Positive EPEs activate seeking motivation that instigates and reinforce approach behavior. Let us explain this idea using the example of seeking behavior for light illumination in rodents. Rodents are attracted to brief illumination of light, which has not been associated with any reward^classic^: When a rat or mouse is placed in a chamber equipped with a lever switch and a lightbulb, the animal explores about the chamber environment including the lever. The animal incidentally depresses the lever and produces a brief light illumination, an event that leads to learning to respond on the lever that produces the light illumination (Keller et al., 2014; Kish, 1966; Stewart and Hurwitz, 1958). Accordingly, novel environment instigates exploratory behavior, and salient light illumination further activates seeking motivation, which in turn instigates seeking behavior for the stimulus. A seeking response that has resulted in a light illumination is reinforced, and rodents learn to make the response again and again as long as the stimulus produces an EPE. As rodents repeatedly experience the stimulus, EPEs will decrease to the point that the exposure no longer activates seeking motivation or behavior. Such explanation can be applied to other observations: Rodents are attracted more to novel contexts and stimuli than familiar ones, as shown by context- and object-choice tasks (Bardo et al., 1989; Ennaceur, 2010); monkeys spend more time gazing at novel stimuli than familiar stimuli (Daddaoua et al., 2016; Ghazizadeh et al., 2016).

Like salient and novel stimuli, uncertainty, i.e., anticipated EPEs, can instigate and reinforce active seeking behavior. Hungry rodents interact more with cues signaling uncertain deliveries of food reward over cues signaling certain outcomes (Anselme et al., 2013). Additionally, monkeys spend more time gazing at stimuli predicting uncertain rewards over certain rewards (Daddaoua et al., 2016; Ghazizadeh, Griggs, and Hikosaka, 2016). Given choices, monkeys often prefer choosing uncertain choices over certain choices associated with rewards^classic^ (Monosov et al., 2015), even if selecting uncertain choices results in fewer overall rewards^classic^ (McCoy and Platt, 2005; Monosov et al., 2015; O’Neill and Schultz, 2010). Likewise, some people enjoy gambling such as playing cards and betting on horse races. Such activities may be instigated and reinforced by uncertainty, i.e., the anticipation of EPEs associated with such activities. In summary, these observations, involving novelty and uncertainty, are consistent with the idea that the detection and anticipation of EPEs can elicit seeking motivation, which instigates and reinforces seeking behavior.

### 2.4. Insights from music perception

Research on music perception provides important insights into the relationship between EPEs and seeking motivation. First, research suggests that the intrinsic reward value of music is tied to the anticipation of upcoming chords and chord sequences (Huron, 2006; Salimpoor et al., 2015). This idea is consistent with our presently proposed perspective, which explains music reward as follows: The anticipation and detection of EPEs concerning chords and chord sequences stimulate and reinforce listening (Fig. 1A).

In addition, research on music perception found that upcoming chords and chord sequences that are easy and difficult to predict have little music value (Cheung et al., 2019). In other words, low and high levels of EPEs concerning chords and chord sequences are less effective than the mid-range level of EPEs in stimulating and reinforcing listening. Fig. 1B depicts an inverted U-shaped relationship between EPEs and seeking motivation. Interestingly, in certain circumstances the value of stimuli or contexts that are complex and, thus produce large EPEs, can increase information values after repeated exposure (Berlyne, 1970). Moreover, the complexity of stimuli exposed immediately earlier can affect the seeking value of the next stimuli that the animal encounters (Berlyne and Crozier, 1971). This may mean that the cognitive capacity to handle information has a limit and that highly complex stimuli overload the system, leading to little information value towards an understanding of the environment. Repeated exposure to the environment producing large EPEs perhaps decrease EPEs, resulting in increased seeking motivation for better understanding of the environment.

Our discussion above explains the latent learning phenomenon that we previously mentioned. The anticipation and detection of EPEs activate seeking motivation, which instigates and reinforces exploratory behavior in novel environments, and this process results in learning about environmental stimuli and their relationships in the absence of reward^classic^.

### 2.5. Seeking motivation can persist in familiar environments

Here we elaborate on the role of seeking motivation in familiar environments where seeking behavior may be regulated by the passage of time. The detection of EPEs instigates seeking motivation to explore environments or objects, and through this activity, animals learn about the environment. However, seeking motivation may not diminish in the environment where the animal has repeatedly interacted. Many environments are complex, and their conditions are dynamic, not static; they constantly change. Even in a relatively static laboratory environment, exploratory behavior does not disappear. For example, when rats are placed in a 40 cm × 40 cm chamber for 30 min daily, rats display robust exploratory activities on day 1, and activities become shorter and less robust over the next several days. However, exploratory behavior never disappears over the ensuing days; rats display exploratory behavior at the beginning of each daily session (Ikemoto, 2002). The absence from the environment for a few hours revives seeking motivation. Therefore, a seemingly simple environment appears to offer a rich medium of EPEs.

### 2.6. Seeking motivation in relation to arousal and stress

Seeking motivation could be described as arousal. The term “arousal” has been used to describe states that coordinate central and peripheral activities in the face of stressful conditions, including actual and foreseen challenges. Stress is often discussed, categorically, as the condition that leads to unwanted physiological states (e.g., high blood pressure, muscle tension, and weakened immune system), negative emotion (e.g., anxiety and depression), and substance use disorders (e. g., alcohol abuse and excessive tobacco smoking). In the present paper, we view stress as existing on a continuum that can be influenced by the perception of novelty and uncertainty. Take, for example, hiking on a trail in the woods. Just the physical act of hiking itself induces some level of stress for a person. If the hiking trail is novel to the hiker, the activity imposes an additional level of stress, accompanied by a higher level of seeking motivation. Furthermore, an encounter with a wild bear while hiking on the trail would be considered extremely stressful and could elicit fight-or-flight behavior, which does not involve seeking motivation. The continuum perspective seems to provide a comprehensive view for understanding behavior. Thus, manageable stressors, those located away from the extremes of this continuum, are healthy conditions that drive active seeking motivation.

The concept of arousal is too broad to discuss seeking processes, as the arousal concept is often used in describing the wakefulness state in contrast to the sleep state and the state associated with extreme stress. For example, how does arousal influence feeding or grooming behaviors? What do animals do when arousal is completely diminished? As you will see below, the seeking motivation concept provides more specific answers to these questions. Therefore, our discussion centers on the concept of seeking motivation, a specific form of arousal that may be alternatively labeled as *seeking arousal*.

### 2.7. Artificial, intracranial manipulations and seeking motivation

The neural substrates of seeking motivation can be exogenously activated by intracranial manipulations. Particularly, the procedures referred to as *intracranial self-administration* (ICSA) and *intracranial self-stimulation* (ICSS) are useful in demonstrating that stimulated neural elements are involved in seeking-behavior processes. In ICSA, animals learn to produce seeking responses to administer neuroactive chemicals intracranially into discrete brain regions (Ikemoto and Wise, 2004), and in ICSS, animals similarly learn to respond for intracranial electrical currents delivered into discrete brain sites (Milner, 1989) or photostimulation that can activate or inhibit specific neural populations with optogenetic procedures (Ilango et al., 2014b). These phenomena can be viewed as the products of activating information-seeking process and then goal-seeking process, or goal-seeking process alone (Fig. 1A).

When Olds and Milner (1954) initially discovered the ICSS phenomenon, such stimulation was thought to induce “pleasure” (Olds, 1956) because of its capacity to reinforce behavior. However, additional observations led to an alternative view. The same manipulations that support ICSS or ICSA instigate exploratory behavioral activities, including increased forward locomotion and sniffing (Ikemoto and Panksepp, 1994; Panksepp, 1981a). In addition, such manipulations augment the seeking responses reinforced by other rewards (Gallistel, 1969; Ikemoto, 2010). Moreover, abused substances, especially psychomotor stimulants, produce not only reinforcing effects, but also behavioral activation effects, and these effects are attributed to their capacity to activate midbrain DA neurons (Wise and Bozarth, 1987). These observations suggest that the manipulations supporting ICSS or ICSA activate neural networks regulating seeking behaviors (Ikemoto, 2010; Ikemoto and Panksepp, 1999; Panksepp, 1982). Therefore, the present paper considers the ICSS and ICSA as the phenomena that arise from the activation of seeking motivation, which instigates and reinforces seeking behavior.

### 2.8. Proposed neural substrates

**Fig. 2.**
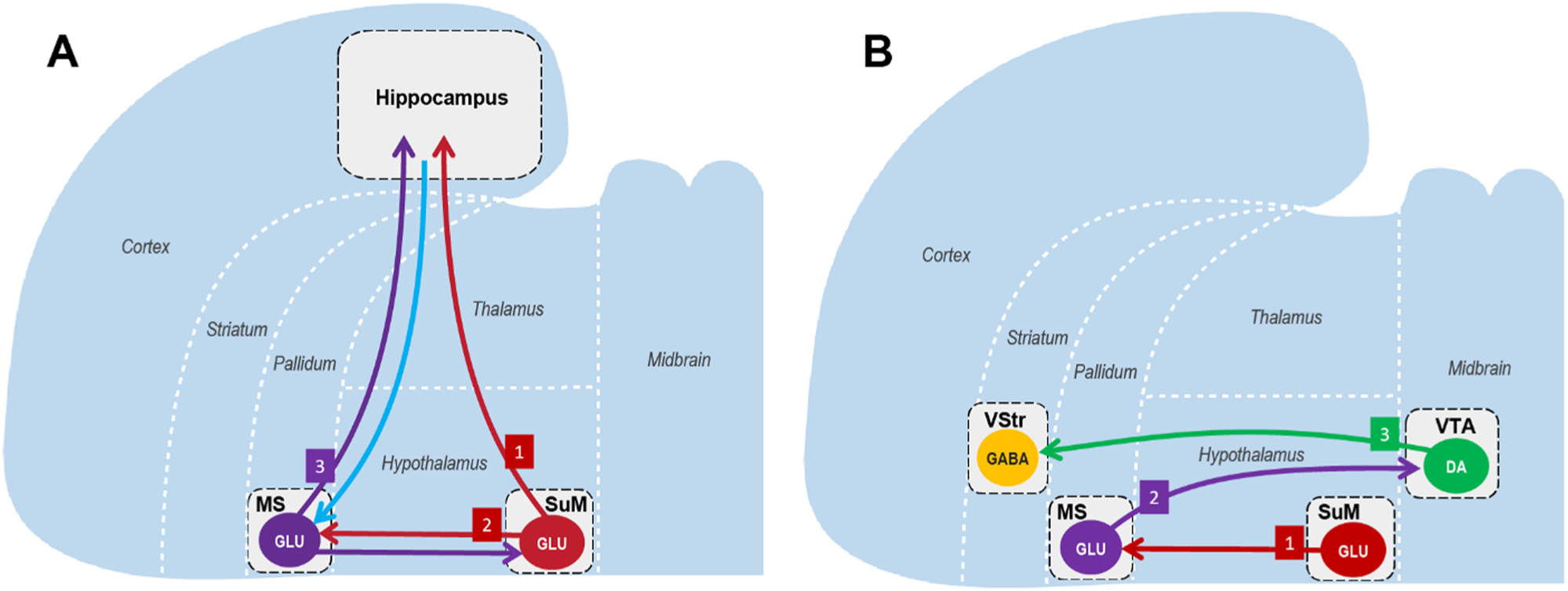
Cognitive and behavioral components of seeking motivation. **A**. The neural substrates mediating the cognitive component of seeking motivation consists of (1) SuMn-to-Hipp, (2) SuMn^Glu^-to-MS, and (3) MS-to-Hipp pathways. In addition, the MS receives input from the Hipp, and the SuM receives input from the MS. **B**. The neural substrates mediating the behavioral component of seeking motivation consists of (1) SuMn^Glu^-to-MS, (2) MSn^Glu^-to-VTA, and (3) VTAn^DA^-to-VStr pathways. Abbreviations: DA, dopaminergic; GABA, gamma-aminobutyric acidergic; Glu, glutamatergic; Hipp, hippocampal formation; LS, lateral septal nuclei; MS, medial septal nucleus; SuM, supramammillary region; VStr, ventral striatum; VTA, ventral tegmental area.

We have discussed that seeking motivation coordinates and regulates (A) *cognitive processes for attention, perception, acquisition, storage, and consolidation concerning environmental information* and (B) *seeking behavior.* We propose two sets of neural pathways that play a fundamental role in seeking motivation (Fig. 2), and central to each pathway is the supramammillary region (SuM). We argue that function (A) depends on (1) SuM neurons (SuMn) projecting to the hippocampal formation (Hipp) (SuMn-to-Hipp) and (2) SuMn projecting to the medial septal area (MS) and (3) then to the Hipp (SuMn-to-MS-to-Hipp) (Fig. 2A). In addition, function (B) depends on (1) glutamatergic (Glu) neurons in the SuM projecting to the MS (SuMn^Glu^-to-MS), (2) glutamatergic MS neurons projecting to the ventral tegmental area (VTA) (MSn^Glu^-to-VTA), and (3) VTA DA neurons projecting to the ventral striatum (VStr) (VTAn^DA^-to-VStr) (Fig. 2B). Note that we do not claim that these pathways are exclusively involved in said functions.

## 3. Supramammillary circuits in seeking motivation

**Fig. 3.**
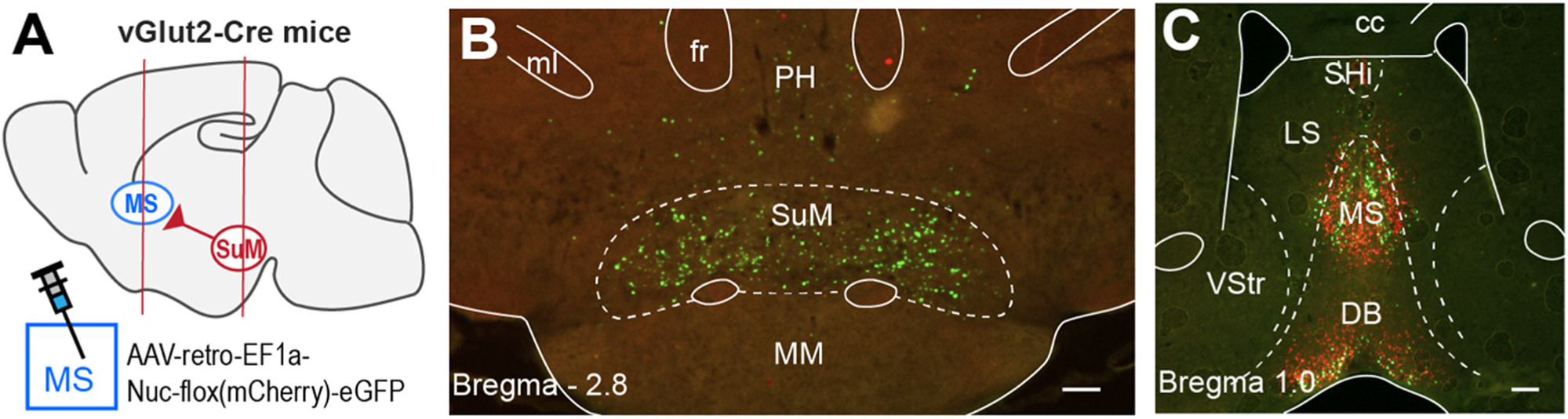
The supramammillo-septal projection. The experiment described in (A) produced results (B, C) suggesting that the SuM-to-MS pathway is primarily glutamatergic. A. The AAV-retro-Nuc-flox(mCherry)-eGFP retrogradely labels Cre-containing soma with eGFP while Cre-negative soma with mCherry. vGlut2-Cre mice received vector injections into the MS. B. SuM neurons are labeled with eGFP, but not mCherry. C. The same injection produced both eGFP and mCherry labeling at the injection sites and in the medial horizontal limb of the DB, which projects to the MS. Abbreviations: cc, corpus collosum; fr, fasciculus retroflexus; LS, lateral septal nuclei; MM, medial mammillary nucleus; ml, medial lemniscus; MS, medial septal nucleus; PH, posterior hypothalamic nucleus; SHi, septohippocampal nucleus; SuM, supramammillary region; VStr, ventral striatum. Scale bars = 200 μm. Figure is adapted from Kesner et al. (2021).

**Fig. 4.**
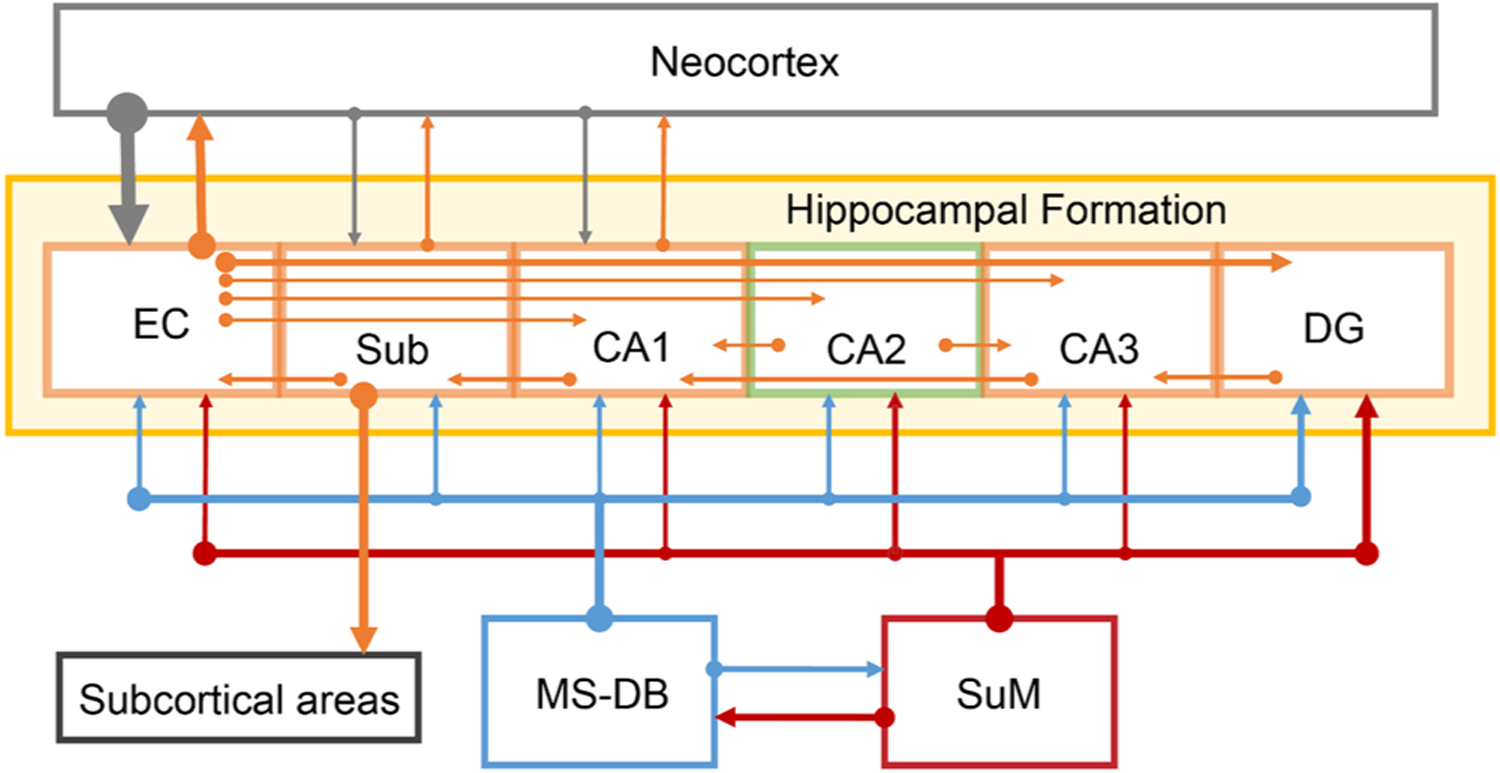
The hippocampal formation and connectivity. Abbreviations: EC, entorhinal cortex; DG, dentate gyrus; MS-DB, medial septal nucleus-diagonal band of Broca; Sub, subiculum; SuM, supramammillary region.

The SuM is a posterior hypothalamic structure located just dorsal to the mammillary body (MB) and anterior to the VTA (Fig. 3). The SuM extensively projects to the septohippocampal system (Vertes, 1992) (Fig. 4). The extensive septohippocampal projections of the SuM underscore the importance of the SuM in regulating the septohippocampal system.

We serendipitously discovered the role of the SuM in reward-seeking behavior. While investigating the functional heterogeneity of VTAn^DA^ in seeking behavior using ICSA procedures, we found that rats self-administer infusions of AMPA directly into the SuM (Ikemoto et al., 2004). This structure had received little attention with respect to reward or reward-seeking behavior, although a study in the 1950 s showed that electrical stimulation of this structure supports ICSS in rats (Olds and Olds, 1958). Our group found that intra-SuM infusions of other excitatory pharmacological agents reinforce behavior, including the GABA_A_ receptor antagonist picrotoxin (Ikemoto, 2005), and nicotine (Ikemoto et al., 2006) – a key substance contained in tobacco that is reinforcing and widely abused. The latter finding implicates the SuM in nicotine addiction. We also found that DA receptor blockade readily attenuates self-administration of intra-SuM AMPA and that intra-SuM AMPA increases extracellular concentrations of DA in the VStr (Ikemoto et al., 2004). We initially had no explanation for how the stimulation of SuM AMPA receptors resulted in increased VStr DA release since we were unaware of the structural relationship between SuM neurons (SuMn) and VTAn^DA^-to-VStr at that time. These initial findings led us to investigate the circuit mechanisms through which the stimulation of SuMn reinforces seeking behavior.

Summarized below are the findings from our recent study (Kesner et al., 2021) supporting that the SuMn^Glu^-to-MSn^Glu^-to-VTAn^DA^ circuit participates in seeking motivation (Fig. 2B):

1. Optogenetic photostimulation of SuMn^Glu^, but not GABA or DA neurons, expressing channelrhodopsin-2 (ChR2), an excitatory opsin, reinforces seeking behavior.
2. Stimulation of SuMn^Glu^-to-MS reinforces seeking behavior.
3. Rats learn to self-administer AMPA along the midline of the septal area, but not the diagonal band of Broca (DB), suggesting that the excitation of neurons in the MS or its vicinity reinforces seeking behavior.
4. Importantly, noncontingent injections of intra-septal AMPA increase seeking behavior reinforced by the 1-s presentation of a salient visual stimulus (bright light), suggesting that increased Glu transmission in the MS increases information-seeking behavior.
5. Stimulation of MSn^Glu^, but not GABA or cholinergic neurons, reinforces seeking behavior.
6. SuMn^Glu^ monosynaptically excite MSn^Glu^.
7. Stimulation of SuMn^Glu^-to-MS increases the signals of GCaMP (a genetically encoded protein that increases fluorescence upon Ca^2+^binding) expressed selectively in VTAn^DA^, suggesting that SuMn^Glu^-to-MS modulates VTAn^DA^.
8. Stimulation of MSn^Glu^ or MSn^Glu^-to-VTA increases the signals of VStr dLight (a protein that fluoresces upon binding DA), indicating that MSn^Glu^-to-VTA modulates DA neuron activity.
9. Pretreatment with DA receptor antagonists decreases seeking behavior reinforced by intra-septal AMPA injections, or by the stimulation of SuMn^Glu^-to-MS.
10. MSn^Glu^ project to the VTA (Fuhrmann et al., 2015) and form synaptic contacts with VTAn. The excitation of MSn^Glu^-to-VTA reinforces seeking behavior. Moreover, the levels of seeking behavior reinforced by stimulation of MSn^Glu^-to-VTA positively correlate with VStr dLight signals driven by such stimulation.

These results suggest that SuMn^Glu^-to-MS modulate MSn^Glu^-to-VTA, which in turn modulate VTAn^DA^-to-VStr and reinforce seeking behavior. Therefore, we suggest that the SuMn^Glu^-to-MSn^Glu^-to-VTAn^DA^ circuit participates in regulating seeking motivation. Importantly, we argue that this circuit is particularly important in coordinating both information-seeking and goal-seeking behaviors.

Note that rodents engage in compulsive-seeking behavior reinforced by brain stimulation, i.e., ICSS, in a stable environment for hours. This observation suggests that ICSS does not depend on acquiring new information, as indicated in Fig. 1A. Therefore, we suggest that the two subsystems can work independently while working together in seeking behavior as (A) a cognitive subsystem and (B) a behavioral subsystem, and that the activation of the motivational subsystem is sufficient to support ICSS without EPEs generated by the cognitive subsystem (Figs. 1 and 2). Below we expand upon the idea that the cognitive aspect of information-seeking behavior is importantly assisted by the hippocampal formation, while the behavioral aspect involves the midbrain DA system, each with functional connectivity to the supramammillo-septal circuit.

## 4. Hippocampal formation and seeking behavior

The hippocampal formation is important for processing environmental information for perception, memory, and prediction (Zeidman and Maguire, 2016). It receives highly processed, multimodal sensory information from cortical regions, including sensory information from olfactory, visual, and auditory cortices, goal-related information from the prefrontal cortex (PFC), and emotion-related information from the amygdala (Amaral and Lavenex, 2007). Thus, it appears to be suited for integrating and comparing incoming information. In general, cortical input reaches the hippocampal formation through the entorhinal cortex, then is further processed by the dentate gyrus, CA3, CA1, subiculum, and back to the entorhinal cortex, in this sequence (Fig. 4) (Amaral and Lavenex, 2007). Although the intrinsic hippocampal circuitry is largely unidirectional, it contains both serial and parallel projections. Nearly all regions of the hippocampal formation receive input from the MS-DB and the SuM (Fig. 4).

Such organization allows the hippocampal formation to integrate multimodal information (Behrens et al., 2018; Gray and McNaughton, 2003; Whishaw and Wallace, 2003) for cognitive mapping (O’Keefe and Nadel, 1978), episodic memory (what, where, and when information for encoding, storing, consolidating, and recalling) (Eichenbaum, 2017; Rolls, 2013), and the detection of novelty (Kumaran and Maguire, 2007, 2009). In addition, it is important in imagining the future, which involves recalling past events and remapping and realigning information for predicting future outcomes (Cheung et al., 2019; Julian and Doeller, 2021; Okuda et al., 2003; Rigoli et al., 2019; Schacter et al., 2012). Such processes must be critical for the generation of EPEs. Thus, the hippocampal formation is an important cognitive component that guides information- and goal-seeking behaviors.

Note that EPEs occur at the levels of synapses, microcircuits, and macrocircuits and that the hippocampal formation is important in high-order EPEs as its connectivity suggests. High-order EPEs depend on the integration of information coming from multiple brain regions. It is particularly important for so-called associative novelty. O’Keefe and Nadel (1978) offered a memorable example of associative memory: “My wife was found in my bed with my best friend.” Novelty does not lie in wife, bed, or best friend, but the combination of the three.

Seeking-related operations of the hippocampal formation are reflected by the occurrence of high-frequency hippocampal theta oscillations (HTO), which are prominent during environmental interaction but not consummatory behaviors (Buzsaki, 2002; Whishaw and Vanderwolf, 1973). HTO reflect essential hippocampal operations of acquiring environmental information for cognitive maps and episodic memory (Buzsaki and Moser, 2013; Eichenbaum et al., 1999; McNaughton and Corr, 2018; Squire, 1992). HTO and related functions are modulated by both MS-DB and SuM, as discussed below.

## 5. MS-DB and seeking behavior

### 5.1. MS-DB neurons respond to saliency and uncertainty

Accumulating evidence suggests that MS-DB neurons play important roles in the cognitive aspect of information-seeking behavior. First, MS-DB neurons critically modulate HTO, as the disruption of MS-DB activity results in diminishing HTO (Winson, 1978). Consistently, the disruption of MS-DB produces deficits in spatial navigation and spatial and episodic memory (Givens and Olton, 1994; Hagan et al., 1988; M’Harzi and Jarrard, 1992; Okada and Okaichi, 2010). Second, MS-DB neurons play an important role in encoding environmental salience. MS-DB neurons respond to salient sensory stimuli in rodents, including visual, auditory, and somatosensory modalities (Hayat and Feldman, 1974; Segal, 1974; Zhang et al., 2018b). Particularly, MSn^Glu^ respond to loud noise (Zhang et al., 2018b). Similarly, MS-DB neurons respond to salient place contexts associated with reward^classic^ in monkeys (Kita et al., 1995; Nishijo et al., 1997). Third, MS-DB neurons respond to uncertainty. Some septal neurons of monkeys increase firing rates in response to conditioned stimuli (CS), signaling uncertain reward outcomes (Monosov and Hikosaka, 2013; Monosov et al., 2015). Specifically, a group of neurons located in the anteromediodorsal part of the septal area display increased activity when reward outcomes are uncertain but not when reward outcomes are certain (Monosov and Hikosaka, 2013). Another population in the MS-DB responds more diversely to stimuli indicating reward uncertainty and certain and uncertain aversive outcomes (Monosov et al., 2015). Therefore, considerable evidence supports the idea that MS-DB neurons are important in the cognitive aspect of information-seeking behavior, including the detection and anticipation of EPEs.

### 5.2. MSn coordinate behavior and cognition in information seeking

As discussed above, HTO are an observable metric reflecting essential activities in seeking behavior in rodents and, thus, are useful windows into the mechanisms underlying seeking motivation. Another observable variable useful in understanding possible neural mechanisms for seeking motivation is locomotor activity, which enables the host to travel in space for environmental information. In fact, the activities of both locomotor activity and HTO are coordinated; MSn^Glu^ modulate both locomotor activity and HTO. First, locomotor speed positively correlates with both the frequency and power of HTO, and HTO increase just before the onset of locomotion (Bender et al., 2015; Green and Arduini, 1954; McFarland et al., 1975; Morris and Hagan, 1983; Vandecasteele et al., 2014; Vanderwolf, 1969; Whishaw and Vanderwolf, 1973). Second, MSn^Glu^ activity increases just before the onset of locomotion and positively correlates with locomotor speed (Fuhrmann et al., 2015). Third, selective optogenetic stimulation of MSn^Glu^ at theta frequencies is sufficient to induce HTO, initiate locomotor activity, and increase locomotor speed in a frequency-dependent manner (Fuhrmann et al., 2015). MSn^Glu^ coordinate locomotion and HTO by regulating the activities of cholinergic and GABA neurons within the MS (Robinson et al., 2016). Together, these findings suggest that these MS-DB cholinergic, GABA, and Glu neurons form a set of local circuits to coordinate HTO and locomotion.

Note that locomotor activity and HTO appear to be modulated by different MSn projections. The projections from the MS to the hippocampus modulate HTO, thereby information integration, as discussed above. In particular, MSn^Glu^ send speed-related information to the medial entorhinal cortex of the hippocampal formation (Justus et al., 2017). For the regulation of movements, MS projections to sub-cortical areas appear to be important. The stimulation of MSn^Glu^ projecting to the preoptic area, but not to the hippocampus, increases locomotor activity (Zhang et al., 2018a). In addition, the MSn^Glu^-to-VTAn^DA^ circuit likely contributes to locomotor activity as this circuit modulates the activity of VTAn^DA^-to-VStr (Kesner et al., 2021), a well-established circuit for controlling locomotor activity and motivation (Ikemoto, 2007; Ikemoto and Panksepp, 1999; Wise, 2004).

Such coordinating role of MS is consistent with the aforementioned finding that the administration of AMPA into the MS increases seeking responses reinforced by the presentation of salient visual stimuli in rats (Kesner et al., 2021).

## 6. SuMn and seeking behavior

SuMn are important in arousal, salience, and hippocampal-dependent information processing. Synthesis of these functions is characterized best in seeking motivation. In particular, SuMn coordinates the activities of other structures that modulate behavior and cognition during information seeking. The SuM interacts closely with the septohippocampal complex in this role.

### 6.1. SuMn^Glu^ modulate HTO via the MS-DB

Although SuMn lesions disrupt HTO only in limited conditions (McNaughton et al., 1995; Pan and McNaughton, 2004; Thinschmidt et al., 1995), the SuM is involved in generating HTO. SuMn transform tonic signals from brainstem regions to a rhythmic signal; this signal is delivered to the MS, which, in turn, relays these signals to the hippocampus (Bland and Oddie, 1998; Kirk, 1998; Kocsis and Vertes, 1994; Pan and McNaughton, 2004). First, inactivation of regions rostral to the SuM modifies the amplitude but not the frequency of reticular-elicited HTO, while inactivation of regions caudal to the SuM affects both frequency and amplitude (Kirk and McNaughton, 1993). Second, the stimulation of SuMn drives HTO (Bland and Oddie, 1998; Kirk, 1998; Pan and McNaughton, 2004; Pedersen et al., 2017; Vertes and Kocsis, 1997), while the inhibition of SuMn decreases the frequency of HTO in freely moving rats (Pedersen et al., 2017; Saji et al., 2000). Moreover, lesions of SuMn produce behavioral deficits similar to those observed from hippocampal lesions (Pan and McNaughton, 2002).

### 6.2. SuMn^Glu^ modulate vigilance states and locomotor activity

Chemogenetic stimulation of SuMn^Glu^ increases wake time and HTO, whereas chemogenetic inhibition of SuMn^Glu^ decreases wake time and the HTO during rapid eye movement (REM) sleep (Pedersen et al., 2017). These effects on vigilance states, i.e., wakefulness and REM sleep (a sleep state where brain activity is similar to active wakefulness) are also consistent with the seeking motivation hypothesis. A lack of seeking motivation results in diminished interest in interacting with the environment, which promotes resting behaviors, including sleep. By contrast, increased seeking motivation is accompanied by increased environmental activities, which prolong wakefulness. In addition, SuMn inhibition induced by microinjections of GABA receptor agonists decreases locomotor activity (Kesner et al., 2021; Ma and Leung, 2007). Conversely, SuMn activation induced by local injections of the GABA_A_ receptor antagonist picrotoxin robustly increases locomotor activity (Shin and Ikemoto, 2010). A more recent study showed that the firing rates of SuM_N_ are highly correlated with locomotor speed in mice and that optogenetic excitation and inhibition of SuM_N_ initiate and diminish locomotor activity, respectively (Farrell et al., 2021).

### 6.3. The activation of SuMn is associated with novelty, uncertainty, and seeking behavior

Research involving c-Fos as a marker for neural activation suggests that SuMn are activated by novel stimuli and uncertainty. c-Fos is strongly induced in SuMn by exposure to the following conditions: novel environments (Wirtshafter et al., 1998); taste cues associated with sickness (Yasoshima et al., 2005); contexts paired with a foot shock (Beck and Fibiger, 1995); odors associated with predators (Day et al., 2004); swim and restraint stress (Cullinan et al., 1995); contexts that allow hungry rats to anticipate food (Le May et al., 2019); and appetitive tasks that require spatial working memory (Vann et al., 2000). As such conditions demand attention and, possibly, actions for further information, these findings are consistent with the idea that SuMn regulate information-seeking processes.

In addition, a link has been established between SuM c-Fos and seeking behavior. Lateral hypothalamic stimulation (Arvanitogiannis et al., 1997) and intra-VTA carbachol injections (Ikemoto et al., 2003) induce c-Fos in SuMn. These manipulations reinforce seeking behavior (Arvanitogiannis et al., 1997; Ikemoto and Wise, 2002) and instigate sniffing and locomotor activity (Ikemoto and Panksepp, 1994; Ikemoto et al., 2003; Miliaressis and LeMoal, 1976), which may reflect seeking motivation. Similarly, intra-SuM picrotoxin injections induce c-Fos, instigate locomotor activity, and reinforce seeking behavior (Ikemoto, 2005; Shin and Ikemoto, 2010).

While SuMn play an important role in hippocampal functions via the MS-DB, a recent report demonstrates that SuMn modulate novelty detection through direct hippocampal projections (Chen et al., 2020). In this study, SuMn projecting to the dentate gyrus increase activity in response to contextual novelty more than social novelty, while SuMn projecting to the CA2 increase activity in response to social novelty more than contextual novelty. Moreover, optogenetic manipulations of SuM-to-dentate gyrus and -CA2 projections alter behavioral responses to contextual and social novelties, respectively (Chen et al., 2020). The results of the study have two notable implications. First, while the unidirectional intrinsic circuitry (Fig. 4) suggests sequential processing of information arriving at the entorhinal cortex, the hippocampal formation integrates qualitatively distinct information depending on the region. Second, different SuM-to-hippocampal pathways are recruited depending on the type of novel stimuli. It is important to examine whether similar functional distinction exists in SuM-to-MS-DB pathways, because, as discussed above, MS-DB-to-hippocampus, -to-preoptic area, and -to-VTA are involved in HTO, locomotor activity, and seeking behavior, respectively.

### 6.4. Unlike VTAn^DA^, SuMn do not selectively respond to reward^classic^ or CS^reward^

**Fig. 5.**
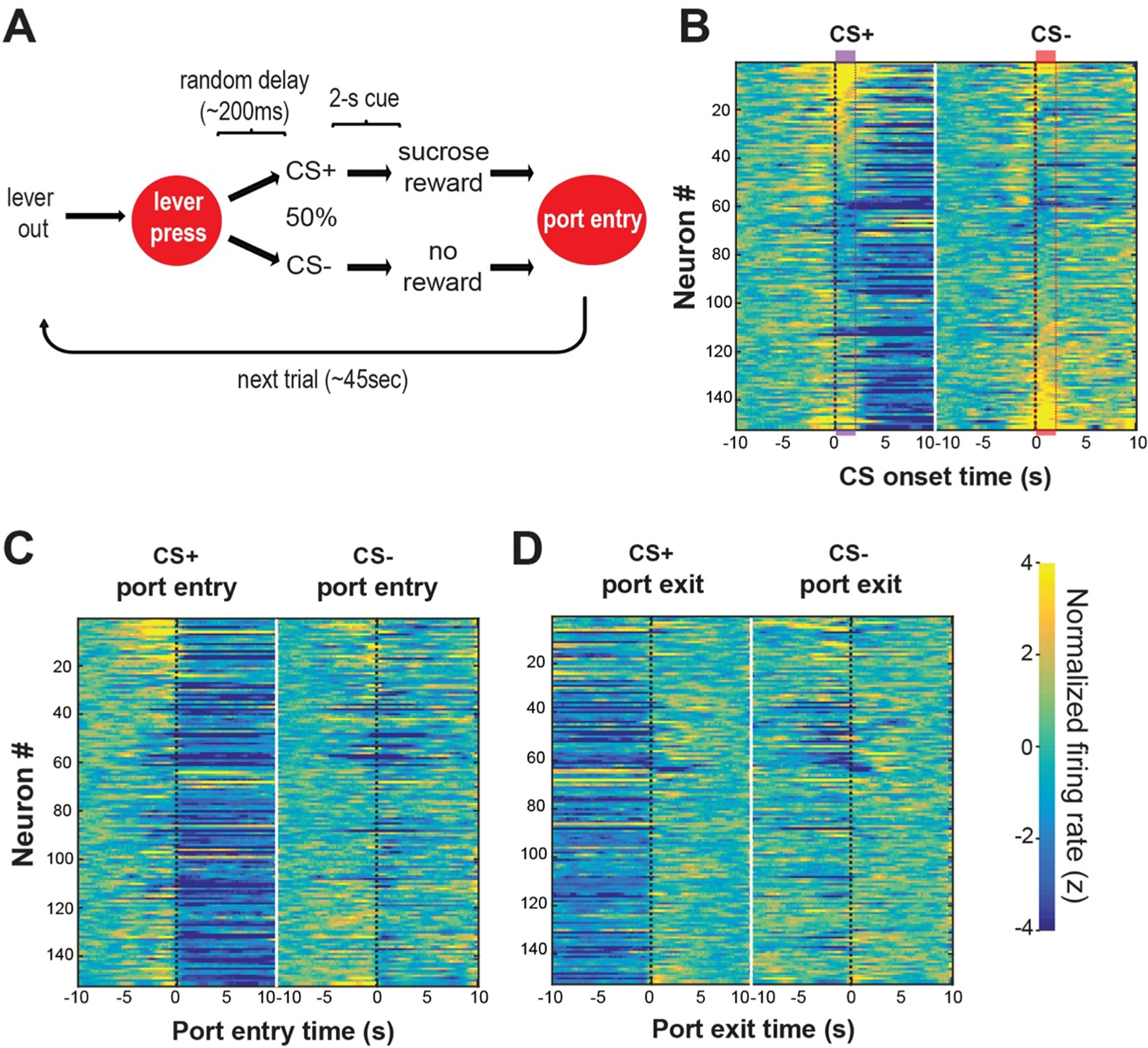
SuM neurons do not respond selectively between CS^reward^ (CS+) and CS^no-reward^ (CS-), but decrease activity during consummatory behavior. A. Diagram showing operant sucrose-seeking procedure with cue discrimination. B-D. Heatmaps of normalized firing rates of SuM neurons during the sucrose-seeking procedure. SuM neuron activities are presented with the reference point (time 0) set at the onset of CS+ or CS- events (B), nose poke entry (C), and nose poke withdrawing (D). Neurons are arranged in the same sequence from top to bottom across all heatmaps. This figure is adapted from Kesner et al. (2021).

DA neuron activity is known to indicate RPEs, and this property of DA neurons plays a critical role in reinforcement learning (Steinberg et al., 2013). Because the stimulation of SuMn reinforces seeking behavior, it is of interest to determine whether SuMn display RPEs or similarly encoded responses. We investigated whether SuMn selectively respond to reward^classic^ and CS^reward^, using electrophysiology recording procedures in freely-moving mice (Kesner et al., 2021). Mice were trained to lever-press for a sucrose reward (Fig. 5A). After a lever press, mice were presented with one of two tones: one tone signaled the availability of the sucrose solution (CS^reward^), while the other had no programmed consequence (CS^no-reward^). The results were surprising, as we expected to observe a significant proportion of SuM neurons responding to CS^reward^: Only a small population of SuMn differentially respond between CS^reward^ and CS^no-reward^ (Fig. 5B).

Similarly, another study found that SuMn do not display predictive activity related to decision making for reward^classic^ (Ito et al., 2018). Rats trained to run on a T-maze had to choose between two arms alternatively for a reward^classic^. SuM activity did not display any information concerning the prediction of choice between the two arms. However, two other structures, the medial prefrontal cortex (mPFC) and reuniens thalamic nucleus, did display such information. Interestingly, inhibition of SuMn decreased the coherence of activity between these two structures in relation to HTO. Given that SuMn directly project to these structures (Vertes, 1992), this study suggests that during environmental interaction, SuMn do not selectively respond to reward^classic^ but that SuMn coordinate other brain structures’ activity with HTO.

The results of the Ito et al. (2018) and the Kesner et al. (2021) studies are consistent with the c-Fos findings that SuMn do not selectively respond to reward^classic^-related stimuli but more broadly to novelty/salience and uncertainty (i.e., the detection and anticipation of EPEs). These discoveries are consistent with the striking single-unit recording observation that SuMn uniformly decrease activity during reward^classic^ intake (Fig. 5C,D) (Kesner et al., 2021) when the host pays little attention to the environment.

### 6.5. The inhibition of SuMn disrupts seeking behavior, but not reward^classic^-taking behavior

Additional results from the Kesner et al. (2021) study support a role for SuMn during seeking behavior. Mice were trained on the same behavioral procedure used for the electrophysiological experiment discussed above. Before testing, mice received an injection of a mixture of the GABA_A_ and GABA_B_ receptor agonists, muscimol and baclofen, into the SuM, to inhibit SuMn. This manipulation disrupted the approach toward the sucrose spout, discrimination between CS^reward^ and CS^no-reward^, and decreased rewards earned and locomotor activity. Although these behavioral deficits are consistent with induced drowsiness or the loss of appetite, the results of our follow-up experiment refute these explanations.

When animals were tested on sucrose consumption that did not depend on instrumental responding, again they displayed decreased locomotor activity, but not the amount of sucrose consumed during the 30-min test. A close examination of the consummatory behavior revealed that when mice received intra-SuM muscimol and baclofen injections, they consumed more sucrose during the first 10 min than when they received saline injections. Because the muscimol/baclofen injection was administered directly into the SuM, the inhibitory action must have been effective immediately, and the increase in sucrose consumption at the beginning of the session argues strongly against the drowsiness or the appetite loss hypotheses. Instead, it is important to consider rodents’ exploratory behavior in incompletely familiar environments. As alluded to above (subsection 2.5), even though rodents explored the test chamber many times in prior days, upon introduction to the test chamber for the day, rodents engage in exploratory behavior about the chamber for the first few minutes before settling down. Therefore, the inhibition of SuMn must have selectively decreased seeking motivation, which interferes with consummatory motivation; the attenuated seeking motivation may have increased the tendency to engage in sucrose-taking behavior over exploratory behavior. These results support that SuM inhibition decreases seeking motivation.

## 7. VTAn^DA^ and seeking behavior

Midbrain DA neurons play a critical role during voluntary behavior, i.e., cortex-driven approach and avoidance, by modulating thalamocortico-basal ganglia processes (Alexander et al., 1986; Haber, 2016; Ikemoto et al., 2015). Particularly, VTAn^DA^-to-VStr are known to modulate stimulus-stimulus and stimulus-response parings and vigor of goal-seeking behavior (Ikemoto, 2007; Ikemoto and Panksepp, 1999). Below, we first discuss the heterogenous roles of midbrain DA neurons and their role in salient and novel stimuli. Then we propose a view that VTA DA neurons, which are known to respond to reward^classic^ and CS^reward^ and indicate RPEs, are better characterized to respond to any type of reward (i.e., rewards^universal^) and indicate EPEs (Fig. 1).

### 7.1. Heterogenous responses of midbrain DA neurons

Midbrain DA neurons have been extensively investigated during reinforcement learning, a process where animals use RPEs to acquire reward^classic^ or avoid punishment (Schultz et al., 1997). However, midbrain DA neurons respond to the environment in a heterogeneous manner. We briefly describe the heterogenous responses of DA neurons. First, some DA neurons respond to movements (Barter et al., 2015; Coddington and Dudman, 2018; Dodson et al., 2016; Howe and Dombeck, 2016; Wang and Tsien, 2011a). Second, a recent study suggests that the heterogeneity of DA neurons emerges as tasks get more complex (Engelhard et al., 2019). Important determining factors considering DA neuron heterogeneity include location and connectivity (Parker et al., 2016). Noxious stimuli consistently excite DA neurons located in the dorsolateral part of the substantia nigra pars compacta, which projects to the dorsolateral striatum or the tail of the striatum (Lerner et al., 2015; Matsumoto and Hikosaka, 2009; Menegas et al., 2017; Schultz and Romo, 1987). DA neurons located outside the dorsolateral part respond variably to aversive stimuli (Brischoux et al., 2009; Coizet et al., 2006; Gore et al., 2014; Guarraci and Kapp, 1999; Jo et al., 2018; Mantz et al., 1989; Wang and Tsien, 2011b).

VTAn^DA^ respond to both reward and aversive stimuli (Brischoux et al., 2009; Guarraci and Kapp, 1999; Jo et al., 2018; Wang and Tsien, 2011b). These differential responses are explained, in part, by their projection sites. Aversive stimuli excite 65% of VTAn^DA^ projecting to the mPFC (Mantz et al., 1989). In addition to mPFC projecting neurons, VTAn^DA^ projecting to the ventromedial and ventrolateral subregions of the nucleus accumbens respond to aversive stimuli (Badrinarayan et al., 2012; Yuan, 2019). However, the same subregions respond to reward as well. Therefore, DA neurons projecting these subregions appear to be involved in salience rather than aversion. This idea based on terminal DA release data should be interpreted cautiously because of possible dissociation between DA release in the terminal regions and cell body firing (Mohebi et al., 2019).

### 7.2. What do phasic responses of DA neurons mean?: Beyond reward^classic^ prediction error

Particularly relevant findings with respect to the present thesis are the following three types of DA neuron phasic responses: First, DA neurons respond to novel sensory stimuli that have not been paired with reward^classic^ (Horvitz, 2000; Redgrave and Gurney, 2006; Schultz, 1998; Wang and Tsien, 2011b). Second, DA neurons increase firing rates upon stimuli predicting advanced information about future reward^classic^ (Bromberg-Martin and Hikosaka, 2009). That is, DA neurons respond to mere information about potential reward^classic^. These stimuli are salient because of their association with CS^reward^ and CS^no-reward^. Third, DA neurons respond to uncertain reward deliveries. DA neurons display preferred responses toward CS signaling riskier delivery over safer delivery of reward^classic^ (Stauffer et al., 2014). Moreover, DA neurons display ramping activity during CS that signal uncertain delivery of reward^classic^ until the potential time of reward^classic^. This ramping activity is absent for a CS signaling either 100% or 0% delivery of reward^classic^ (Fiorillo et al., 2003). Note that the ramping activity is not considered a phasic response, but rather a tonic activity change. In sum, DA neurons respond to not only reward^classic^ and CS^reward^, but also to other rewards, including novel sensory stimuli, mere information, and uncertainty.

In addition, DA neurons can respond to information concerning non-reward^classic^. Active avoidance behavior is suggested to be a form of approach behavior – approach toward “safety”, and mediated by, in part, DA neurons: Increased DA activity instigates and reinforces active avoidance, while decreased DA activity diminishes active avoidance (Ikemoto and Panksepp, 1999). Recent evidence provides stronger support for this view. The presentation of CS paired with a foot shock (CS^aversive^) instigates freezing behavior, i.e., a passive avoidance. However, repeated trials may lead to the emergence of active avoidance behavior. This transformation in behavior coincides with the change in DA release in the accumbens core. When animals display passive avoidance behavior (i.e., freezing), core DA levels do not increase upon CS^aversive^; when the same animals later display active avoidance behavior, core DA levels increase upon CS^aversive^ (Gentry et al., 2016; Oleson et al., 2012). Thus, these studies suggest that DA neurons are involved in transforming passive avoidance behavior into active avoidance behavior and support the view that DA neurons respond to information concerning non-reward^classic^, to regulate active seeking behavior. Note that because these studies measured DA release in the accumbens core, it is not clear whether the presentation of CS^aversive^ alters DA neuron firing rates as avoidance behavior is transformed from passive to active.

### 7.3. DA neurons display prediction errors of salient stimuli

We suggest that phasic responses of DA neurons are better characterized as prediction errors of rewards^universal^, which includes rewards^classic^, CS^reward^, and novel or salient stimuli associated with information seeking motivation. Indeed, phasic DA signal as generalized prediction error has been suggested (Gardner et al., 2018), and internal models such as that described in Fig. 1 have been proposed to explain such activity (Suri, 2001). Let us first discuss how arbitrary the concept of rewards^classic^ is in terms of RPE. It is difficult to determine the exact moment of the receipt of rewards^classic^ and to distinguish CS^reward^ from actual rewards^classic^ (Wise, 2002). For example, when do animals detect a food reward? When they see it, touch it, taste it, or finally digest it? The recognition of a reward^classic^ appears to consist of a series of stimulus-stimulus sequences with adaptive responding during each step: Sight instigates reaching out for it; touch instigates initial ingestion; and taste instigates chewing and swallowing it, which gradually provides energy as it gets digested. Therefore, defining the absolute moment of the receipt of rewards^classic^ may be arbitrary. An important implication of this analysis is that RPEs indicate errors for the magnitude and timing of rewards^universal^. Speaking generally, DA neurons respond when a potentially important stimulus (i.e., rewards^universal^) occurs unpredictably, and if something else can predict the unpredicted important stimulus, then DA neurons begin responding to that thing.

This function of DA neurons is sufficient to explain the biphasic DA-neuron response, which consists of a fast component signaling mere saliency of sensory stimuli and a slow component associated with reward^classic^ (Schultz, 2016). Although the two components of DA signals have been suggested to be functionally distinct (Schultz, 2016), both components appear to signal the same, rewards^universal^, which could guide goal-seeking behavior. Accordingly, the first component indicates DA neurons’ capacity to respond to salient stimuli, i.e., rewards^universal^ that have not been predicted or identified in reference to internal models; the second component indicates CS^reward^, i.e., rewards^universal^ that are known as the predictors of potentially important stimuli by internal models, but not predicted. The biphasic DA neuron response is explained by computational time required for perception. The perception of single-modality sensory stimuli requires fewer neural relays and is thereby processed faster than CS^reward^. For example, novel visual stimuli may evoke excitation of DA neurons through signals from the superior colliculus (Redgrave and Gurney, 2006), which receives visual information directly from the retina (Sefton and Harvey, 2004). On the other hand, conditioned visual stimuli, by definition, depend on the retrieval of prior experience, which is represented by bits and pieces of memory stored in multiple cortical regions (Bar, 2004); therefore, such retrieval and synthesis delay the perception of CS^reward^. Although novel sensory stimuli can affect postsynaptic neurons more quickly than CS^reward^, there is no evidence that the apparent differences in timing are interpreted differently by postsynaptic target neurons. Instead, the first component of the biphasic response may have the same function as the second. As mentioned above, phasic DA responses can be triggered by the presentation of novel sensory stimuli, including visual stimuli. Salient visual stimuli, which are not associated with reward^classic^, can instigate and reinforce seeking behavior, a process that depends on VTAn^DA^-to-VStr (Shin et al., 2010). Therefore, post-synaptic cells may not be affected differently by the two components of the biphasic response, and this analysis supports the idea that phasic responses of DA neurons are better characterized as prediction errors of rewards^universal^, which include novel stimuli and rewards^classic^.

### 7.4. DA neurons modulate behavioral responses instigated by novelty and uncertainty

The aforementioned findings are largely correlational in nature, showing strong relationships between phasic increase in DA neuron activity and the occurrences of salient stimuli and uncertainty, although evidence for the latter is thin at this time (Fiorillo et al., 2003; Stauffer et al., 2014). However, accumulating evidence suggests causal role of DA neurons in modulating seeking behavior instigated by novel stimuli and uncertainty. Pharmacological manipulations used to increase DA activity enhance seeking responses for CS^reward^ by increasing the salience of CS^reward^ (Berridge and Robinson, 1998), and such action of DA takes place in the VStr (Ikemoto and Panksepp, 1999). Notably, VStr DA modulates seeking motivation for salient stimuli. As alluded above, focal injections of amphetamine, which stimulates the release and blocks the uptake of dopamine, into the VStr increase DA concentration and increase seeking responses for unconditioned, salient visual stimuli in a DA receptor type 1 (D1R)- and type 2 (D2R)-dependent manner (Shin et al., 2010). Consistently, systemic injections of the D2R antagonist haloperidol decrease preference for novel stimuli over familiar ones in rodents (Bardo et al., 1989). Moreover, the novelty-seeking personality trait correlates with VTA/substantia nigra activity in response to novel stimuli in humans (Krebs et al., 2009).

In addition, DA modulates seeking behavior under uncertainty. Administration of DA receptor agonists and antagonists can respectively decrease and increase seeking behavior under uncertainty. For one, the administration of pramipexole, a non-ergot D2R agonist used to treat Parkinson’s disease, increases pathological gambling behavior (i.e., a seeking behavior under uncertainty) in a treatment-dependent manner (Dodd et al., 2005; Driver-Dunckley et al., 2003). Similarly, the D2R agonist cabergoline decreases the subjective cost of responding to uncertain choices in healthy humans (Le Heron et al., 2020).

Uncertainty-driven behaviors observed during pharmacological manipulation of DA activity may be controlled by the striatum, particularly the VStr, and the PFC. Rodent research suggests that VStr DA activity appears to modulate seeking behavior under uncertainty (Piantadosi et al., 2021). Typical procedures compare two choices between a small, certain reward^classic^ and a large, uncertain reward^classic^. Intra-VStr injections of D2R agonists, which inhibit D2R-expressing neurons, increase choices for large, uncertain reward outcomes (Zalocusky et al., 2016). Conversely, intra-VStr D2R antagonists, which activate D2R-expressing neurons, decrease large, uncertain choices (Cocker et al., 2012). Notably, one study demonstrated similar behavioral effects with pharmacological manipulations of VStr D1Rs, but not D2Rs (Stopper et al., 2013). Moreover, phasic activation of VStr D2R-expressing neurons immediately after choices decreases subsequent choices for large, uncertain reward outcomes (Zalocusky et al., 2016). By contrast, lesions or pharmacological inhibition of VStr neurons decrease responses for choices linked to large, uncertain outcomes (Cardinal and Howes, 2005; Floresco et al., 2018; Mai et al., 2015; Stopper and Floresco, 2011). In humans, polymorphisms within the D2R gene predict striatal D2R density and are associated with reinforcement learning using punishment, or “No-Go learning.” Individuals with two copies of the D2R T-allele have greater striatal D2 receptor density and a greater tendency to avoid choices linked with negative consequences (Frank et al., 2009, 2007). Moreover, individuals with two copies of the DARPP-32 T-allele, which affects D1R-mediated striatal synaptic plasticity, have a greater tendency to choose responses associated with positive consequences (Frank et al., 2009). Thus, while VStr DA plays an important role in behavior under uncertainty, the specific VStr subregions and the respective roles of D1Rs and D2Rs during behavior under uncertainty have not been established. Importantly, early evidence indicates that VStr DA signals interact with cortical inputs arriving from the PFC, amygdala, and hippocampus (Piantadosi et al., 2021).

Increasing evidence suggests that the PFC is involved in decision-making based on uncertainty levels in humans. Polymorphisms within the Catechol-O-methyltransferase gene affect DA levels in the PFC, which are important for decision-making based on whether other choices might produce better outcomes than the status quo. Carriers of a particular allele have a greater propensity for explorative decisions (Blanco et al., 2015; Frank et al., 2009, 2007; Kayser et al., 2015). Indeed, modeling PFC control of behavioral functions suggests its involvement in learning and forecasting the probable outcomes of actions (Alexander and Brown, 2011).

In summary, while DA responses to CS^reward^ have been suggested to functionally differ from DA responses to novel stimuli, postsynaptic target neurons may not differentiate these two stimuli. Thus, in addition to rewards^classic^ and CS^reward^, DA neurons play an important role in seeking behavior instigated by novel stimuli and uncertainty.

## 8. Differential roles of VTAn^DA^ and SuMn^Glu^ in seeking behavior

Let us clarify the different roles of VTAn^DA^ and SuMn^Glu^. VTAn^DA^ participate in producing seeking behavior toward goals by interacting with the thalamo-cortico-basal ganglia system (Ikemoto et al., 2015). VTAn^DA^ appear to be essential in developing stimulus-stimulus and stimulus-response associations concerning goal-seeking and invigorating goal-seeking behavior (Ikemoto, 2007); thus, VTAn^DA^ are overwhelmingly involved in seeking rewards^universal^, toward which the animal should learn to approach. While little is known about the role of SuMn^Glu^ in seeking behavior, the available evidence (Section 6) suggests that SuMn^Glu^ modulate general environmental interaction instigated by novelty and uncertainty. In other words, SuMn^Glu^ are responsible for coordinating cognitive and behavioral systems for environmental interaction. In this regard, SuMn^Glu^ may be important in both active seeking and passive avoidance behavior and may respond to a broad range of EPEs compared to VTAn^DA^ (Fig. 1B). As discussed in Section 3, the stimulation of the SuMn^Glu^-to-MSn^Glu^ pathway instigates active seeking behavior, and we consider the MSn^Glu^-to-VTA pathway as an interface between SuMn^Glu^ and VTAn^DA^ that shapes information-seeking behavior into goal-seeking behavior where the goal is information. By contrast, stimulation of the SuMn^Glu^-to-paraventricular thalamic nucleus pathway may be involved in avoidance behavior as it elicits aversion (Kesner et al., 2021). Such an idea needs to be examined by future research.

## 9. Implications

### 9.1. Neural network of active seeking behavior

The aforementioned pathways involving SuMn-to-Hipp, SuMn^Glu^-to-MS-to-Hipp, and SuMn^Glu^-to-MSn^Glu^-to-VTAn^DA^ imply that the flow of signals among these structures are hierarchical and linear; however, adaptive behavior most likely depends on dynamic interactions between many brain structures. For example, in addition to midbrain DA neurons and septal neurons (Monosov and Hikosaka, 2013; Monosov et al., 2015), evidence indicates that neurons in other brain regions also respond to uncertain rewards, including the anterior and posterior cingulate cortex, orbital frontal cortex, striatum, zona incertus, and lateral periaqueductal gray (McCoy and Platt, 2005; O’Neill and Schultz, 2010; White et al., 2019). Therefore, an extended network is most likely responsible for controlling seeking behavior.

**Fig. 6.**
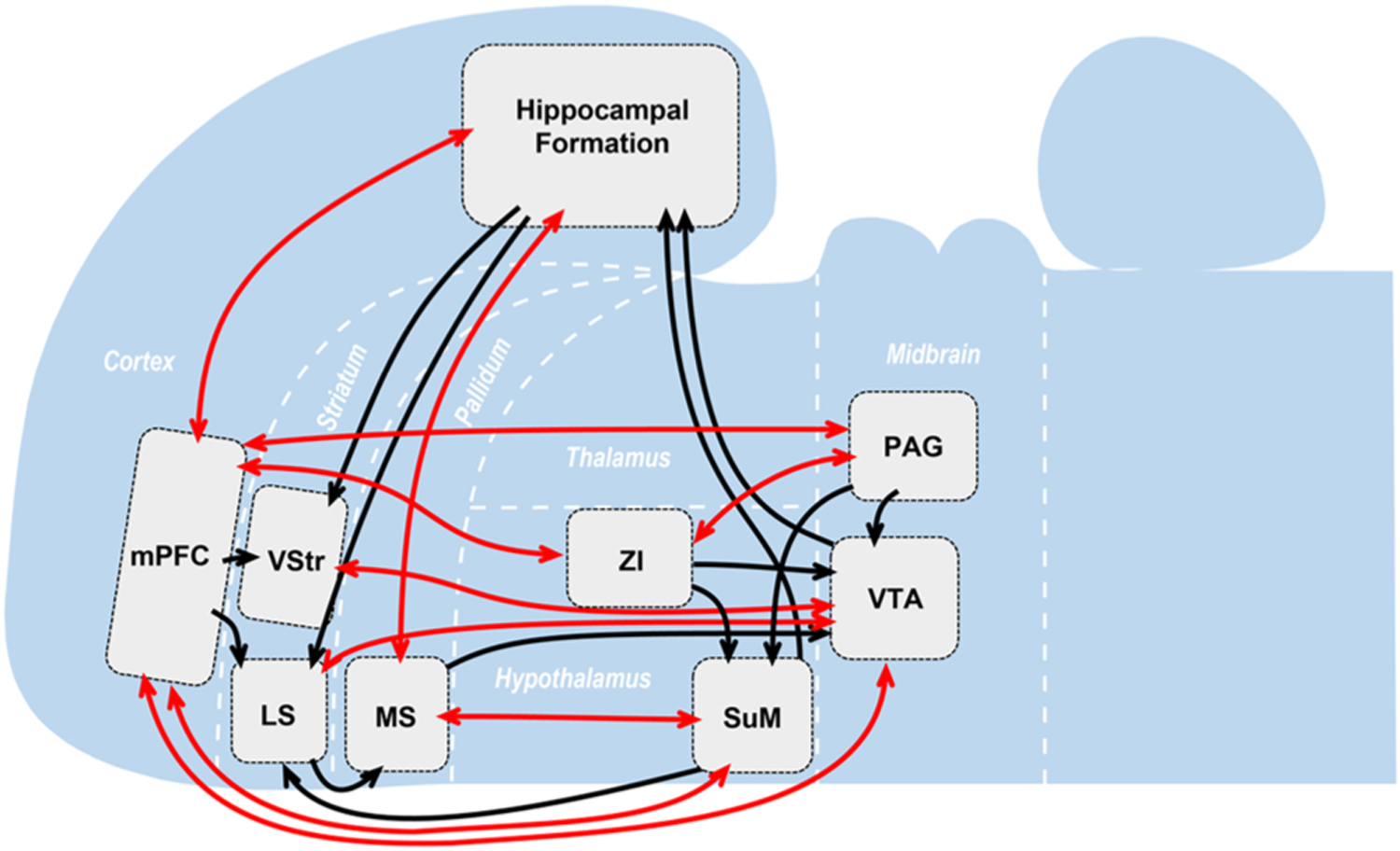
Key structures of the network involved in seeking motivation. The red lines indicate reciprocal neural connectivity, while the black lines indicate predominantly unidirectional connectivity. In particular, the interaction with the hippocampus is suggested to play a key role in information-seeking behavior. Note that this diagram by no means represent all important structures that participate in information-seeking behavior. Abbreviations: LS, lateral septal nuclei; MS, medial septal nucleus; PAG, periaqueductal gray; PFC, prefrontal cortex; SuM, supramammillary region; VStr, ventral striatum; VTA, ventral tegmental area; ZI, zona incerta.

In particular, a recent study identified that the mPFC-to-zona incerta (ZI)-to-periaqueductal gray (PAG) circuit modulates novelty-seeking behavior (Ahmadlou et al., 2021). In addition, the study’s findings suggest that there may be at least two levels of information-seeking behavior. The activation and deactivation of the mPFC-to-ZI pathway or ZI-to-PAG pathway respectively increase and decrease high, but not low, level information-seeking behavior, and the level of information-seeking behavior is correlated with the activation level of the neurons of these pathways (Ahmadlou et al., 2021). Note that the SuM-to-MS-to-VTA may be involved in both levels of information-seeking behavior since the inhibition of the neurons of these pathways disrupts mere locomotor activity. However, we suspect that the SuM-to-MS-to-VTA pathway belongs to the same seeking-behavior network as the mPFC-to-ZI-to-PAG pathway. First, the ZI projects to the SuM and VTA (Fig. 6). Second, the PAG projects to the SuM and VTA. Third, the mPFC is reciprocally linked with the SuM, VTA, and hippocampal formation (Fig. 6). It is of interest to investigate how these regions interact during seeking behavior.

### 9.2. Nicotine addiction

As mentioned in the introduction, micro-infusions of nicotine into the SuM reinforce behavior (Ikemoto et al., 2006). This finding raises the question of whether the SuMn^Glu^-to-MSn^Glu^-to-VTAn^DA^-to-VStr circuit is involved in nicotine reward and addiction. In addition, it will be important to determine whether nicotinic acetylcholine transmission within the SuM is involved in regulating behavioral responses to salient stimuli and uncertainty. To this end, nicotinic acetylcholine receptors containing the β2 subunit have been implicated in controlling behavior under uncertainty (Addicott et al., 2013; Naude et al., 2016) and, importantly, the β2 subunit is expressed in the SuM (Wada et al., 1989). Moreover, nicotine self-administration in rats diminishes with the removal of salient stimuli accompanied by nicotine infusions (Caggiula et al., 2001). In other words, the co-presentation of salient stimuli is essential in maintaining nicotine self-administration in rats. Such an observation begs the question of whether the SuMn^Glu^-to-MSn^Glu^--to-VTAn^DA^-to-VStr circuit can provide a mechanistic explanation for how salient stimuli promotes nicotine self-administration.

### 9.3. Psychopathology

The SuMn^Glu^-to-MSn^Glu^ may be a critical subcortical mechanism for curiosity, which we believe is an active information-seeking behavior. In this light, dysregulation of the SuMn^Glu^-to-MSn^Glu^-to-VTAn^DA^-to-VStr circuit may lead to psychopathological conditions: Hyperactivity of this circuit may contribute to pathological behaviors under uncertainty, such as uncontrolled gambling. Conversely, hypoactivity of this circuit may lead to the motivational disorder known as abulia and apathy, characterized as the lack of motivation for voluntary behavior or active information-seeking behavior.

## 10. Coda

We have proposed two sets of neural pathways focusing on the SuM, arguing that SuMn-to-Hipp and SuMn^Glu^-to-MS-to-Hipp pathways are important in the cognitive aspect of information-seeking behavior, including the detection and anticipation of EPEs, and that the SuMn^Glu^-to-MSn^Glu^-to-VTAn^DA^ pathway plays an important role in instigating and reinforcing seeking behavior. It is important to investigate how these pathways participate in different aspects of seeking behavior and motivation. We believe that this perspective paper provides a useful framework for future research on seeking motivation.

## Abbreviations:

CS^reward^: conditioned stimulus associated with reward
CS^no-eward^: conditioned stimulus associated with no programmed consequence
CS^aversive^: conditioned stimulus associated with aversive stimulus
ChR2: channelrhodopsin-2
D1R: dopaminergic receptor type 1
D2R: dopaminergic receptor type2
DA: dopamine
DB: diagonal band of Broca
EPE: environment prediction error
Glu: glutamatergic
Hipp: hippocampal formation
HTO: hippocampal theta oscillations
ICSS: intracranial self-stimulation
MS: medial septal area
PAG: periaqueductal gray
REM: rapid eye movement
reward^classic^: classic reward
reward^universal^: any type of reward
RPE: reward prediction error
SuM: supramammillary region
SuM_N_: supramammillary neurons
VTA_N_^DA^: ventral tegmental doopaminergic neurons
VStr: ventral striatum
ZI: zona incerta
